# Tertiary hyperparathyroidism with ectopic mediastinal parathyroid adenoma in a patient with chronic kidney disease and cardiovascular complications: a case report

**DOI:** 10.1186/s12902-025-02160-3

**Published:** 2026-01-02

**Authors:** Mohammed M. Salahaldin, Azzam Zrineh, Abdullah Abu Keshek, Hamza Karmi, Montaser Badran, Mohammad Bourini

**Affiliations:** 1 Faculty of Medicine, Al-Quds University, Jerusalem, Palestine; 2 Department of Internal Medicine, Al Makassed Hospital, Al-Quds University, Jerusalem, Palestine

**Keywords:** Tertiary hyperparathyroidism, Chronic kidney disease, Ectopic parathyroid adenoma, Mediastinal mass, Tc-99m sestamibi, SPECT/CT, Cardiovascular complications.

## Abstract

**Background:**

Hyperparathyroidism includes conditions marked by excessive secretion of parathyroid hormone (PTH), resulting in disruptions in calcium-phosphate metabolism. In individuals with chronic kidney disease (CKD), differentiating secondary hyperparathyroidism from tertiary hyperparathyroidism is frequently challenging due to the overlapping biochemical and clinical characteristics. Ectopic mediastinal parathyroid adenomas are uncommon and complicate localisation and therapy further.

**Case presentation:**

We present a 66-year-old female with chronic kidney disease, hypertension, and ischaemic heart disease, who had increasing dyspnoea initially attributed to heart failure. Laboratory assessment indicated PTH 238.8 pmol/L, corrected calcium 2.545 mmol/L, phosphorus 1.389 mmol/L, and vitamin D 39.8 nmol/L, suggesting advanced hyperparathyroidism. The primary diagnostic issue was distinguishing tertiary hyperparathyroidism from secondary hyperparathyroidism in the context of chronic kidney disease (CKD). Localisation was accomplished with Tc-99m sestamibi SPECT/CT, revealing a 3 × 2 cm anterior mediastinal lesion consistent with an ectopic parathyroid adenoma. Echocardiography demonstrated significant concentric left ventricular hypertrophy and a large pericardial effusion. Definitive surgical treatment consisting of bilateral neck exploration with thoracoscopic excision of the anterior mediastinal parathyroid gland was recommended following stabilization. However, after counseling, the patient declined surgical intervention and was managed conservatively with ongoing nephrology and endocrinology follow-up.

**Discussion:**

This case highlights the diagnostic complexity of tertiary hyperparathyroidism in CKD and the crucial role of multimodal imaging in localising ectopic adenomas. Integrating biochemical, radiological, and clinical findings is essential for accurate diagnosis and timely surgical planning.

**Conclusion:**

This case highlights how modest biochemical irregularities and unusual cardiopulmonary symptoms can obscure ectopic parathyroid disease, emphasising the importance of maintaining a high index of suspicion to guide optimal clinical decision-making.

**Clinical trial number:**

Not applicable.

**Supplementary Information:**

The online version contains supplementary material available at 10.1186/s12902-025-02160-3.

## Introduction

Hyperparathyroidism encompasses a spectrum of disorders characterised by excessive parathyroid hormone (PTH) secretion, resulting in disturbances of calcium-phosphate homeostasis with diverse systemic manifestations. The condition is clinically classified into primary, secondary, and tertiary forms, each with distinct pathophysiological mechanisms and clinical implications. Primary hyperparathyroidism arises from autonomous overactivity of one or more parathyroid glands, most commonly due to a solitary adenoma, and typically presents with hypercalcaemia [1]. In contrast, secondary hyperparathyroidism (SHPT) represents a compensatory physiological response to chronic hypocalcaemia, particularly prevalent in patients with chronic kidney disease (CKD), where impaired phosphate excretion and diminished vitamin D activation drive persistent parathyroid stimulation [2].

CKD affects over 10% of the global population, with secondary hyperparathyroidism emerging as one of its most significant complications [3]. The pathophysiological cascade involves hyperphosphataemia, hypocalcaemia, elevated fibroblast growth factor-23 (FGF23), and reduced 1,25-dihydroxyvitamin D3 levels, all contributing to parathyroid gland hyperplasia and increased PTH production [3, 4].

The majority of patients with CKD exhibit secondary hyperparathyroidism, generally accompanied by hypocalcaemia or normocalcaemia. However, a minority of these cases progress to tertiary hyperparathyroidism (THPT), which is clinically defined by the autonomous overproduction of PTH and the resultant hypercalcaemia [1]. THPT arises as a complication in approximately 8% of patients with SHPT [5]. Although the pathology in the majority of THPT cases is demonstrated as hyperplasia affecting all four glands, single or double adenomas, and very infrequently, carcinomas, have also been documented as underlying causes [5, 6].

The majority of hyperfunctioning parathyroid glands are found in the cervical region, typically adjacent to the thyroid gland, consistent with their embryologic development. However, due to abnormalities during embryonic migration, 15–20% of these glands are located ectopically, along the path of descent from the third and fourth pharyngeal pouches, including sites such as the thymus, retroesophageal space, and the mediastinum. Importantly, approximately 1–3% of these ectopic cases are situated deep within the thoracic cavity (anterior or posterior mediastinum), requiring a specialized thoracic surgical approach for successful resection [7].

Mediastinal parathyroid glands pose a unique challenge in surgery and are a rare cause of refractory or recurrent hypercalcaemia [8]. While the majority of these glands can be successfully removed through a standard neck incision, those that are larger, deeper, or completely within the mediastinum require direct thoracic access. Historically, this involved highly invasive procedures like sternotomy or thoracotomy, which carried substantial patient morbidity. However, the introduction of video-assisted thoracoscopic surgery (VATS) has improved outcomes by allowing surgeons to retrieve these glands less invasively, guided by advanced localisation tools [8].

The distinction between advanced secondary and early tertiary hyperparathyroidism can be particularly challenging in CKD patients, as biochemical profiles frequently overlap and hypercalcaemia may be subtle or masked by concurrent renal and cardiovascular comorbidities. This diagnostic challenge may be compounded when PTH overproduction originates from ectopic parathyroid tissue. In such cases, imaging becomes essential for precise localisation and assessment of functional activity. Techniques such as Tc-99 m sestamibi SPECT/CT and ¹⁸F-fluorocholine PET/CT have markedly improved the detection of ectopic mediastinal lesions, with the application of combined techniques offering superior diagnostic accuracy [7].

We report a case of a woman with chronic kidney disease who presented with progressive dyspnoea and biochemical evidence of tertiary hyperparathyroidism. Comprehensive evaluation revealed an ectopic anterior mediastinal parathyroid adenoma measuring 3 × 2 cm on computed tomography, confirmed by technetium-99 m sestamibi SPECT/CT. This case contributes to the limited literature by documenting the constellation of tertiary hyperparathyroidism, ectopic mediastinal adenoma, and severe cardiovascular complications in a CKD patient. Specifically, it highlights: [1] the potential for cardiovascular manifestations, particularly pericardial effusion, to dominate the clinical presentation of tertiary hyperparathyroidism; [2] the value of multimodal imaging in localising ectopic parathyroid tissue in complex anatomical locations; and [3] the therapeutic considerations when balancing surgical intervention against significant perioperative risk in patients with advanced CKD and cardiovascular comorbidity.

## Case presentation

A 66-year-old female, non-smoker, married, with a past medical history of chronic kidney disease, hypertension, and ischaemic heart disease, presented with progressive shortness of breath of 14 days duration. She had a background of exertional dyspnoea for the preceding six months, limiting her walking capacity to less than 50 m, which had been attributed to chronic heart failure. Over the two weeks prior to admission, her symptoms worsened, progressing to dyspnoea at rest, orthopnea, and paroxysmal nocturnal dyspnoea. She denied chest pain, palpitations, cough, sputum production, fever, or weight loss.

On examination, the patient appeared in mild respiratory distress. Her vital signs included a blood pressure of 154/89 mmHg, a heart rate of 80 beats per minute, and a respiratory rate of 20 breaths per minute. Chest auscultation revealed bilateral basal crepitations, dullness to percussion over the lower zones, decreased chest expansion, and localised wheezes in the left upper zone. Cardiac examination demonstrated muffled heart sounds and a pansystolic murmur of grade III intensity at the left lower sternal border. Abdominal examination revealed distension with a positive cough impulse at the umbilicus, suggestive of an umbilical hernia. There was trace bilateral pedal oedema, but no cyanosis, clubbing, or palpable lymphadenopathy.

Chronic kidney disease was established clinically and by laboratory results, with the patient having bilaterally small kidneys on imaging, a persistently elevated baseline serum creatinine measured three months prior to admission (221–248 µmol/L [2.5–2.8 mg/dL]), and concomitant anaemia, findings that collectively substantiate a chronic rather than acute renal process. Biochemical investigations further supported this diagnosis, demonstrating a markedly elevated parathyroid hormone (PTH) level of 238.8 pmol/L, corrected serum calcium of 2.545 mmol/L, phosphorus of 1.389 mmol/L, and vitamin D deficiency (39.8 nmol/L). Renal function tests revealed an elevated serum creatinine of 2.8 mg/dL and blood urea nitrogen (BUN) of 23.67 mmol/L, consistent with chronic kidney disease.

Haematological studies demonstrated normocytic normochromic anaemia with haemoglobin of 101 g/L and thrombocytopenia with a platelet count of 120 × 10⁹/L, while the leukocyte count was within normal limits. ESR was elevated at 37 mm/hr. Urinalysis demonstrated proteinuria, microscopic hematuria, and bacteriuria, while urine studies confirmed marked proteinuria.

**Fig. 1:**
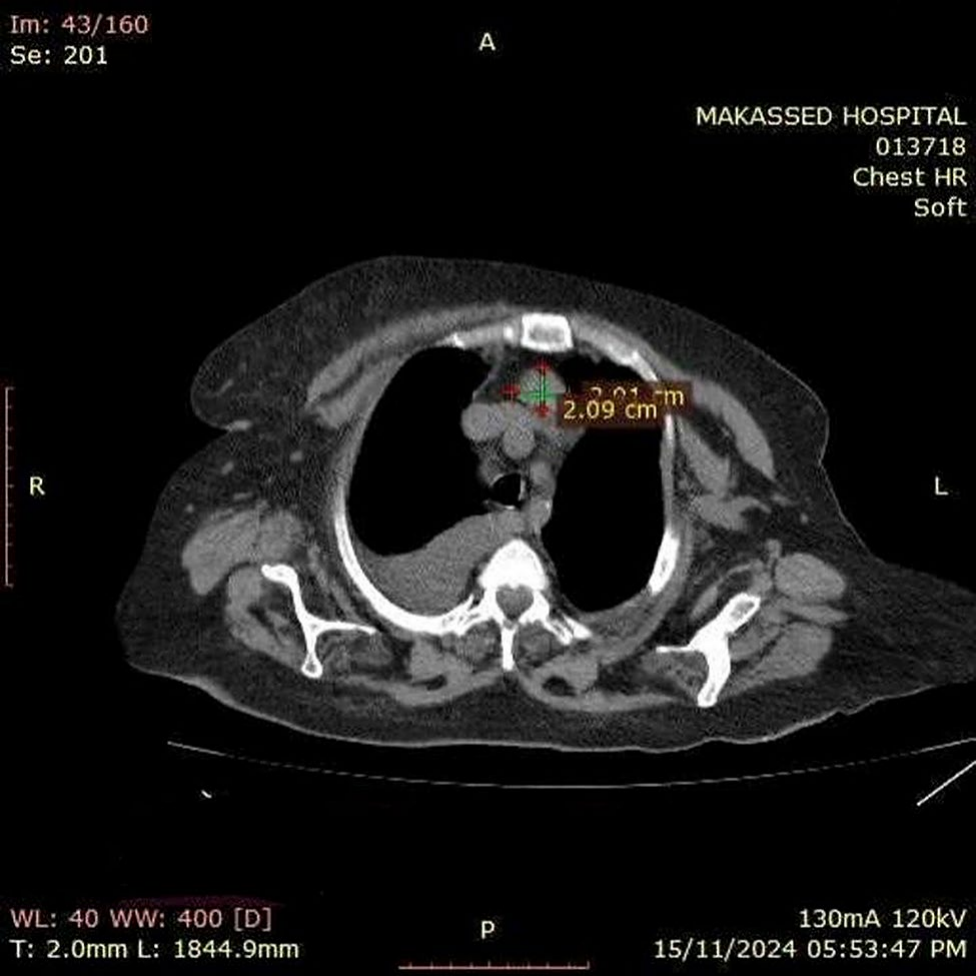
Chest CT scan showing a well-defined anterior superior mediastinal soft tissue mass measuring approximately 2.09 × 2.11 cm, consistent with ectopic parathyroid adenoma as indicated by parathyroid scintigraphy findings

**Fig. 2:**
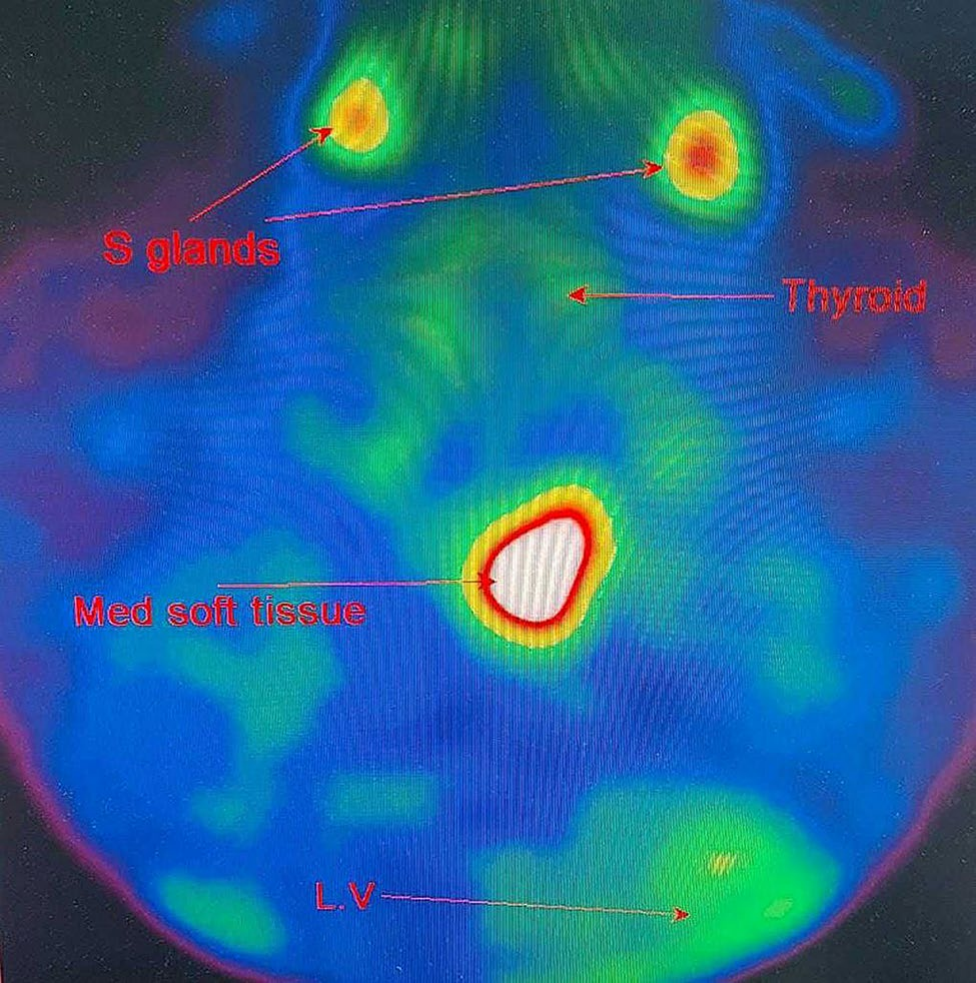
Tc-99m Sestamibi parathyroid scan showing abnormal focal tracer uptake in the anterior superior mediastinum. Findings are consistent with ectopic parathyroid adenoma

Neck ultrasound demonstrated mild thyroid enlargement without nodules and benign-appearing cervical lymph nodes. A chest CT scan revealed a 3 × 2 cm soft tissue lesion in the anterior mediastinum [Figure 1]. A parathyroid sestamibi scan (Tc99m-MIBI) confirmed focal abnormal tracer uptake at the same site, consistent with ectopic parathyroid adenoma [Figure 2]. Abdominal ultrasound showed hepatomegaly (18 cm), echogenic kidneys consistent with chronic kidney disease, bilateral pleural effusion (more prominent on the right), and an umbilical hernia. Echocardiography demonstrated preserved left ventricular systolic function, severe concentric left ventricular hypertrophy, grade I diastolic dysfunction, and severe global pericardial effusion without tamponade, as well as mild mitral and tricuspid regurgitation.

The patient was managed conservatively with oxygen therapy, diuretics, and optimisation of heart failure therapy, which led to symptomatic improvement. Given the biochemical profile and imaging findings, an ectopic parathyroid adenoma located in the anterior mediastinum was diagnosed.

**Fig. 3:**
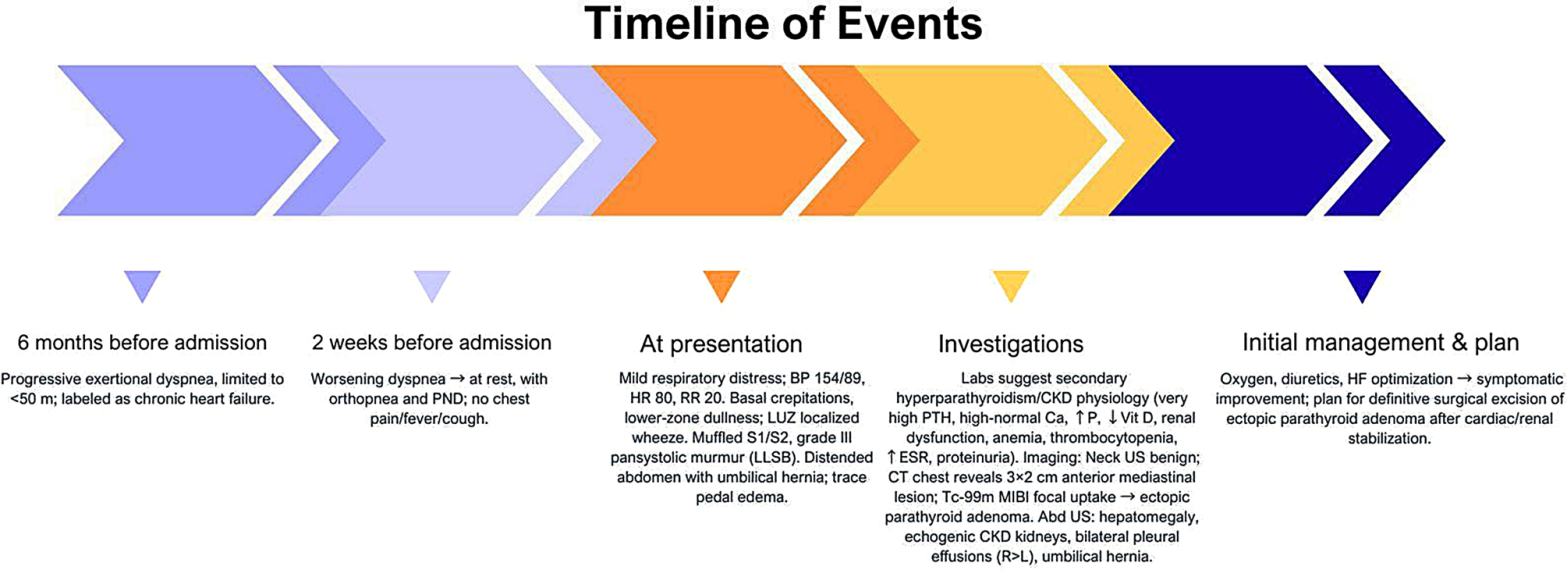
A timeline of events for the reported case

Definitive surgical excision was planned following stabilization of her cardiac and renal status. The recommended surgery in such scenario is bilateral neck exploration with thoracoscopic excision of the anterior mediastinal parathyroid gland. However, the patient had declined operative intervention after multidisciplinary counselling regarding risks, benefits, and expected outcomes. She was therefore managed conservatively with nephrology and endocrinology follow-up, optimization of CKD-mineral and bone disorder therapy, and serial monitoring of serum calcium, phosphate, parathyroid hormone, and alkaline phosphatase. As surgery had not been performed, histopathology and postoperative biochemical outcomes were not available. [Figure 3] represents a timeline of events for this patient.

## Discussion

This case illustrates the diagnostic and therapeutic challenges encountered when tertiary hyperparathyroidism with an ectopic mediastinal parathyroid adenoma presents in a patient with chronic kidney disease and significant cardiovascular comorbidity. The patient’s presentation with progressive dyspnoea, initially attributed to heart failure, exemplifies how autonomous parathyroid disease in CKD can manifest with atypical cardiovascular symptoms rather than the classical skeletal and renal complications traditionally associated with hyperparathyroidism.

### Differential diagnosis

A critical consideration in this case is distinguishing between secondary and tertiary hyperparathyroidism, particularly given the patient’s established chronic kidney disease. Secondary hyperparathyroidism is common in CKD patients, with prevalence increasing from less than 20% in patients with GFR over 60 mL/min/1.73 m² to over 50% in those with GFR less than 30 mL/min/1.73 m² [9]. The pathophysiology involves hypocalcemia, decreased 1,25-dihydroxyvitamin D synthesis, hyperphosphataemia, and FGF23 elevation, all of which stimulate parathyroid hormone secretion [4, 10]. Tertiary hyperparathyroidism represents the natural progression of prolonged secondary hyperparathyroidism, where one or more parathyroid glands undergo autonomous transformation and continue producing excessive PTH despite normalisation or elevation of serum calcium.

In this patient, several features strongly support tertiary hyperparathyroidism. First, the markedly elevated PTH level (238.8 pmol/L) is disproportionate to the modest hypercalcaemia (2.545 mmol/L), a pattern characteristic of tertiary hyperparathyroidism, where prolonged CKD-induced parathyroid stimulation results in autonomous glandular hyperfunction. Second, the patient’s long-standing CKD provides the chronic stimulus necessary for the development of tertiary disease. Third, while imaging demonstrated a solitary adenoma rather than multiglandular hyperplasia, tertiary hyperparathyroidism can present with hyperpalsia of a single gland [5, 11]. The combination of vitamin D deficiency (39.8 nmol/L), elevated phosphorus (1.389 mmol/L), and chronic kidney disease creates the ideal milieu for progression from secondary to tertiary hyperparathyroidism. Tertiary hyperparathyroidism arising in CKD patients may necessitate more extensive parathyroid exploration and consideration of total parathyroidectomy with autotransplantation to prevent persistent or recurrent disease. Furthermore, the cardiovascular manifestations observed in this case may be amplified by the dual contributions of chronic kidney disease and autonomous parathyroid hyperfunction.

### Cardiovascular manifestations: a life-threatening dimension

The cardiovascular complications observed in this patient underscore the growing recognition of cardiac disease as a potentially life-threatening manifestation of hyperparathyroidism in CKD. The combination of chronic kidney disease and autonomous parathyroid hyperfunction creates a particularly high-risk scenario for cardiovascular complications. Parathyroid hormone exerts positive inotropic and chronotropic effects that promote left ventricular hypertrophy [12]. The severe global pericardial effusion without tamponade observed in this case represents an uncommon but clinically significant cardiovascular manifestation, possibly related to calcium deposition in pericardial tissue combined with inflammatory processes. The concurrent severe concentric left ventricular hypertrophy and diastolic dysfunction align with established evidence that PTH plays an important role in maintaining myocardial hypertrophy [13, 14].

### Mediastinal ectopic parathyroid adenomas: localisation and management

Mediastinal ectopic parathyroid adenomas account for only 1–2% of all parathyroid adenomas [15], making this case particularly noteworthy. The 3 × 2 cm size observed is consistent with reported mediastinal adenomas, which tend to be larger than cervical adenomas due to delayed diagnosis. The combination of Tc-99 m sestamibi SPECT/CT with contrast-enhanced CT, as utilized in this case, represents the routinely employed modalities for mediastinal adenoma localisation, while combined techniques offer more diagnostic accuracy [7].

Surgical management of mediastinal ectopic parathyroid adenomas has evolved significantly. Although traditional sternotomy provides excellent exposure and enables direct palpation, video-assisted thoracoscopic surgery (VATS) and robot-assisted thoracoscopic surgery (RATS) have revolutionized the management of mediastinal parathyroid disease [16–18]. The recommendation for definitive surgical excision in this case, following cardiac and renal stabilization, reflects contemporary understanding that surgery represents the only curative treatment.

### Learning points and clinical implications

1. Tertiary hyperparathyroidism should be strongly considered in CKD patients with markedly elevated PTH disproportionate to modest hypercalcaemia, particularly in the setting of long-standing renal dysfunction, vitamin D deficiency, and hyperphosphataemia.
2. Solitary ectopic adenomas can occur in tertiary hyperparathyroidism through autonomous transformation of a single gland, and should not automatically be interpreted as primary disease when clinical context suggests chronic secondary hyperparathyroidism.
3. Cardiovascular manifestations, including pericardial effusion and left ventricular hypertrophy, may dominate the clinical presentation in tertiary hyperparathyroidism and represent the combined effects of CKD and autonomous parathyroid hyperfunction requiring urgent medical stabilization.
4. Distinguishing tertiary hyperparathyroidism in CKD requires careful integration of biochemical, radiological, and clinical data.
5. Minimally invasive surgical approaches represent the current standard of care for mediastinal parathyroid disease when anatomically feasible.

### Limitations and future directions

In this case report, two important limitations must be acknowledged. First, we were unable to obtain postoperative biochemical data or pathology confirmation, as surgery was recommended but declined by the patient. Postoperative PTH levels and histopathological examination would definitively confirm the diagnosis. Long-term follow-up with serial PTH measurements is essential, as persistent or recurrent hyperparathyroidism would strongly support tertiary disease and might necessitate re-exploration for residual hyperplastic glands. Second, the absence of historical PTH values from earlier stages of CKD limits our ability to document the progression from secondary to tertiary hyperparathyroidism.

Future research should focus on developing better biomarker panels and imaging protocols to distinguish tertiary hyperparathyroidism in CKD patients, as current tests often overlap and may delay optimal treatment. Moreover, studies investigating the natural history of secondary-to-tertiary hyperparathyroidism progression could identify predictive factors for autonomous transformation and guide preventive interventions. Furthermore, long-term studies examining cardiovascular reversibility after parathyroidectomy in tertiary hyperparathyroidism would provide insights into optimal surgical timing and help establish evidence-based guidelines for this complex patient population.

## Conclusion

This case underscores the educational importance of identifying unusual biochemical and cardiac manifestations of tertiary hyperparathyroidism in patients with chronic kidney disease. It emphasises the critical role of combining biochemical evaluation with functional imaging, such as Tc-99 m sestamibi SPECT/CT, to resolve diagnostic uncertainty and accurately localise ectopic parathyroid adenomas. Clinically, this case has important educational value, as it reinforces how early identification of ectopic tertiary hyperparathyroidism can directly influence management strategies, guiding timely surgical intervention and improving patient outcomes.

## Supplementary Information

Below is the link to the electronic supplementary material.

Supplementary Material 1

## Data Availability

All data generated or analyzed during this study are included in this published article.
