# Supplementary material for: Tertiary hyperparathyroidism with ectopic mediastinal parathyroid adenoma in a patient with chronic kidney disease and cardiovascular complications: a case report
Source: BMC Endocr Disord. 2026 Jan 2;26:30. doi: 10.1186/s12902-025-02160-3 (PMC12865936; doi:10.1186/s12902-025-02160-3)
Supplement: Supplementary file 1 — Supplementary Material 1 [file 12902_2025_2160_MOESM1_ESM.pdf]

CARE case report guidelines

CARE Checklist of information to include when writing a case report

(cc) BY-NC-ND

Topic	Item	Checklist item description	Reported on Line
**Title**	1	The diagnosis or intervention of primary focus followed by the words "case report"	1-3
**Key Words**	2	2 to 5 key words that identify diagnoses or interventions in this case report, including "case report"	74-76
**Abstract (no references)**	3a	Introduction: What is unique about this case and what does it add to the scientific literature?	42-48
	3b	Main symptoms and/or important clinical findings	49-53
	3c	The main diagnoses, therapeutic interventions, and outcomes	50-63
	3d	Conclusion—What is the main "take-away" lesson(s) from this case?	69-72
**Introduction**	4	One or two paragraphs summarizing why this case is unique (**may include references**)	80-141
**Patient Information**	5a	De-identified patient specific information.	147-155
	5b	Primary concerns and symptoms of the patient.	147-155
	5c	Medical, family, and psycho-social history including relevant genetic information	165-179
	5d	Relevant past interventions with outcomes	N/A
**Clinical Findings**	6	Describe significant physical examination (PE) and important clinical findings.	156-164
**Timeline**	7	Historical and current information from this episode of care organized as a timeline	202-203
**Diagnostic Assessment**	8a	Diagnostic testing (such as PE, laboratory testing, imaging, surveys).	180-189
	8b	Diagnostic challenges (such as access to testing, financial, or cultural)	45-47; 220-231;305-313
	8c	Diagnosis (including other diagnoses considered)	220-231
	8d	Prognosis (such as staging in oncology) where applicable	N/A
**Therapeutic Intervention**	9a	Types of therapeutic intervention (such as pharmacologic, surgical, preventive, self-care)	190-203
	9b	Administration of therapeutic intervention (such as dosage, strength, duration)	190-191
	9c	Changes in therapeutic intervention (with rationale)	N/A
**Follow-up and Outcomes**	10a	Clinician and patient-assessed outcomes (if available)	191
	10b	Important follow-up diagnostic and other test results	200-201
	10c	Intervention adherence and tolerability (How was this assessed?)	N/A
	10d	Adverse and unanticipated events	N/A
**Discussion**	11a	A scientific discussion of the strengths AND limitations associated with this case report	294-303
	11b	Discussion of the relevant medical literature **with references**.	212-276
	11c	The scientific rationale for any conclusions (including assessment of possible causes)	220-276
	11d	The primary "take-away" lessons of this case report (without references) in a one paragraph conclusion	277-293; 321-329
**Patient Perspective**	12	The patient should share their perspective in one to two paragraphs on the treatment(s) they received	N/A
**Informed Consent**	13	Did the patient give informed consent? Please provide if requested 348-351	Yes ☑ No ☐
